# An Effective and Robust Decentralized Target Tracking Scheme in Wireless Camera Sensor Networks

**DOI:** 10.3390/s17030639

**Published:** 2017-03-20

**Authors:** Pengcheng Fu, Yongbo Cheng, Hongying Tang, Baoqing Li, Jun Pei, Xiaobing Yuan

**Affiliations:** 1 Science and Technology on Microsystem Laboratory, Shanghai Institute of Microsystem and Information Technology, Chinese Academy of Sciences, Shanghai 201800, China; fupc@mail.sim.ac.cn (P.F.); cyb@mail.sim.ac.cn (Y.C.); tanghy@mail.sim.ac.cn (H.T.); libq@mail.sim.ac.cn (B.L.); peijun@mail.sim.ac.cn (J.P.); 2 University of Chinese Academy of Sciences, Beijing 100049, China

**Keywords:** target tracking, wireless camera sensor networks, information filter, camera selection, decentralized, sensor fusion

## Abstract

In this paper, we propose an effective and robust decentralized tracking scheme based on the square root cubature information filter (SRCIF) to balance the energy consumption and tracking accuracy in wireless camera sensor networks (WCNs). More specifically, regarding the characteristics and constraints of camera nodes in WCNs, some special mechanisms are put forward and integrated in this tracking scheme. First, a decentralized tracking approach is adopted so that the tracking can be implemented energy-efficiently and steadily. Subsequently, task cluster nodes are dynamically selected by adopting a greedy on-line decision approach based on the defined contribution decision (CD) considering the limited energy of camera nodes. Additionally, we design an efficient cluster head (CH) selection mechanism that casts such selection problem as an optimization problem based on the remaining energy and distance-to-target. Finally, we also perform analysis on the target detection probability when selecting the task cluster nodes and their CH, owing to the directional sensing and observation limitations in field of view (FOV) of camera nodes in WCNs. From simulation results, the proposed tracking scheme shows an obvious improvement in balancing the energy consumption and tracking accuracy over the existing methods.

## 1. Introduction

Among many surveillance functions of Wireless Sensor Networks (WSNs), tracking a moving target in a sensing field is a major one that has wide-spread areas of applications, such as habitat monitoring, traffic monitoring, and intruder tracking [1,2,3]. In target tracking, the current presence of moving targets will be detected by sampling the sensed signals (e.g., light, sound, image, or video) [4]. In recent years, with the price of smart camera dropping rapidly, the development of wireless camera sensor networks (WCNs) has been heavily fostered [5,6]. Hence, a new trend in target tracking is to deploy sensor nodes with smart cameras to capture, process and analyze image data locally and to send extracted information back to the sink node [7]. However, the target tracking in WCNs is greatly different from that in traditional WSNs with respect to camera field of view (FOV), bandwidth consumption, and multimedia data processing [8]. Therefore, much attention should be paid to some special target tracking schemes in WCNs.

Due to the requirement for an uninterrupted and reliable tracking network system, decentralized or distributed tracking approaches are usually preferred much more than centralized solutions in WCNs. Distributed approaches, e.g., [9,10], aim to achieve scalability and high fault tolerance for large networks, where the measurements are maintained in several task nodes across the tracking network [6]. However, much energy consumption is usually required in task nodes in terms of processing information data and communicating with their neighbour nodes due to the consensus algorithms. Hence, decentralized solutions, e.g., [11,12,13,14], may be partial to be adopted for some application situations that use camera nodes with limited energy, since they behave well in balancing energy consumption and resilience to faults. In such solutions, a task cluster will be formed and all cluster nodes will detect the target and process locally. Subsequently they forward information data to the selected CH which fuses different results and acts as the cluster scheduler. Therefore, the decentralized schemes need measurement integration methods (e.g., Kalman filter (KF), information filter (IF), particle filter (PF), etc.) and task cluster selection mechanisms.

The tracking algorithms that depend on linear filters, such as traditional KF and IF, cannot be applied to camera networks, since the target measurements provided by the camera sensors are non-linearly related to each other [15]. The consensus filter is a popular and efficient method to take tracking task in a distributed framework, e.g., [10,16,17], but inappropriate to cluster-based decentralized tracking systems as it requires us to ensure agreement among all neighbouring nodes. In decentralized systems, some variants of information filter such as extend information filter (EIF) [18], cubature information filter (CIF) [19] and square root cubature information filter (SRCIF) [20] are preferred due to the fact that they can distribute the computational burden and be easily extended for decentralized multi-sensor cooperative state estimation. CIF is derived from EIF and cubature Kalman filter (CKF) [21]. SRCIF is the square root version of CIF, where the square root covariances matrix is propagated to make the entire filter robust against round-off errors [19]. In our work, we also employ SRCIF to integrate different measurements because it is numerically stable and robust compared to other filter algorithms [19,20].

WCNs tend to evolve into large-scale networks with limited bandwidth and energy resources, especially for outdoor surveillance applications. Hence, a single target may be viewed simultaneously by a large number of camera nodes. Decentralized solutions that use numerous task nodes could improve tracking accuracy at the expense of large communication overhead as well as high energy consumption, which, however, leads to the reduction of network lifespan. Hence, improving tracking accuracy and prolonging network lifespan are two conflicting requirements in WCNs with limited energy. An efficient way to balance the two requirements requires only a desired number of camera nodes to participate in the tracking task with satisfaction of relevant requirements. Therefore, how to select appropriate task cluster nodes (including the CH) is of critical importance in decentralized target tracking, and subsequently is the main goal of our work.

Bernab et al. [22] present an entropy-based algorithm that dynamically selects multiple camera nodes to balance sensing performance and energy consumption. Additionally, the CH is also dynamically selected using entropies and transmission error rates therein. Nevertheless, entropy is a nonlinear metric and its computation is inappropriate for a decentralized approach. In [23], novel camera activation and CH selection mechanisms that consider transmission errors and use the trace of information matrix as uncertainty metric are developed, where all camera nodes that their rewards overtake the cost will be activated to form the tracking cluster. However, both of the above works assume an omnidirectional sensing model, i.e., the target can be viewed within a circular area whose radius equals to the sensing range of camera nodes. In practical circumstances, they are hard to directly apply to WCNs due to the directional sensing and limitations in FOV of cameras. In [7], the authors propose a surprisal selection method to facilitate the camera nodes to take independent decision on whether their observations are informative or not, which considers the directional sensing nature of camera nodes. However, this method fixes the fusion center (FC) and requires the knowledge of the total number of camera nodes that view the current target.

Network lifetime and tracking accuracy are two main concerns for target tracking in WCNs. Work [4] has proposed and proved that a smaller energy balance metric of a network implies a longer lifetime of the tracking network, given a total amount of energy. Therefore, the balance of energy distribution should be paid much attention when selecting the task camera nodes and their CH. In our work, we propose an efficient and robust multi-sensor decentralized target tracking scheme for WCNs. More specifically, considering characteristics and constraints of WCNs such as directional sensing, limited energy, observation limitations in FOV of cameras and insufficient computational capability, we utilise the following mechanisms to efficiently carry out tracking tasks: (1) a more realistic camera node sensing model; (2) a decentralized SRCIF for fusing different observation results at the CH; (3) an efficient mechanism for selection of task cluster nodes that balances the energy consumption and tracking accuracy; and (4) a mechanism that selects the CH by taking a compromise between the remaining energy and the distance-to-target.

This paper focuses on balancing energy consumption and tracking accuracy in single target tracking in dense WCNs. Note that some problems such as boundary detection, losses of data packets and recovery of the lost target are assumed to be out of the scope of the paper. Our main contributions are:

- Proposing a greedy on-line decision mechanism to select task cluster nodes based on the defined contribution decision (CD) which quantifies the expected information gain and the energy consumption. This mechanism dynamically changes the weight of energy consumption in CD according to the related remaining energy of the node in a current candidate node set.
- Designing an efficient CH selection mechanism that casts such a selection problem as an optimization problem based on the predicted target position and the remaining energy.
- Analysing the probability of a target precisely detected by camera nodes when selecting suitable task cluster nodes and their CH, in consideration of the inaccuracy of the predicted next target position.
- Integrating all proposed mechanisms into a decentralized tracking scheme in order that these mechanisms can be implemented efficiently and steadily.

The rest of this paper is structured as follows. In Section 2, we formulate some tracking problems in WCNs and discuss main system models. Section 3 introduces a decentralized SRCIF algorithm for measurement fusion. The proposed mechanisms of cluster node selection and CH selection are detailed in Section 4 and Section 5, respectively. Section 6 illustrates the decentralized tracking scheme which integrates all proposed mechanisms. In Section 7, we evaluate the proposed mechanisms and compare them with state-of-the-art methods. Section 8 concludes the paper and discuss our future work.

## 2. Problem Formulation and System Models

### 2.1. Problem Formulation and System Overview

**Figure 1:**
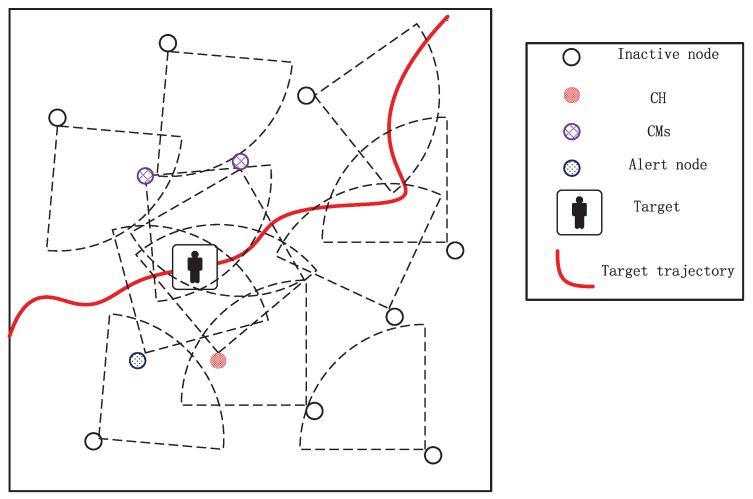
A target tracking scenario in a wireless camera sensor network. The target is viewed by many nodes, but only some of them form the tracking task cluster and the remaining nodes turn into the alert node. In addition, the nodes that could not view the target will turn into the sleep state to save energy.

**Figure 2:**
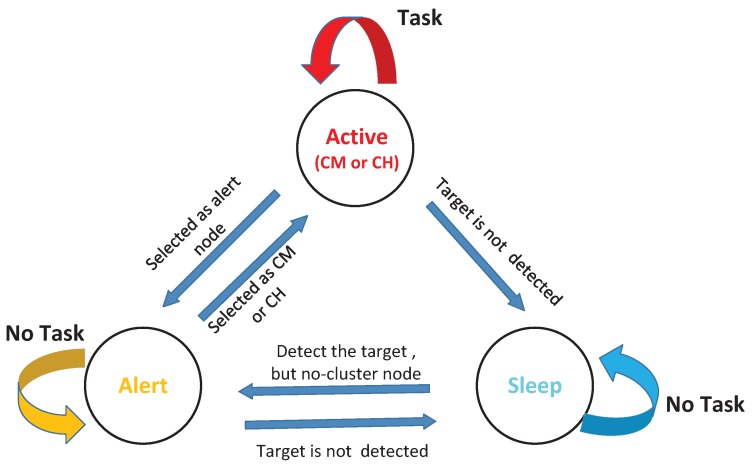
The state transition process model.

As shown in Figure 1, in this work, a single target (e.g., human, animal, low-speed vehicle) and lots of camera sensor nodes are assumed to be located in a 2-D plane network. We consider a dense network consisting of *N* calibrated and cheap camera sensor nodes $C=\{c_{1},c_{1},\cdots ,c_{N}\}$, each of which is assumed to have sufficient capacity to execute simple image processing techniques with limited energy. The task of the camera nodes in the network is to monitor the given environment and to track an object. Additionally, we assume that all sensor nodes are in three states, namely active, alert and sleep state. If a node is a cluster node, it will stay active to track the target in the current timestep. Meanwhile, if a node that could detect that the current target does not belong to the current task cluster, it will turn into the alert state which could be quickly activated if necessary. Those nodes that could not view the current target will be put into sleep state to save energy, but periodically awaken to sense the target. Once they detect the target in their field of view (FOV) at timestep *k*, they will turn into alert state. Note that each node is only in one of the three states during one timestep. Figure 2 describes the relationship of three state models.

While a mobile target is going through the monitored area, some of the camera nodes that are sensing the target will be activated to form a single-hop cluster. Each cluster has a cluster head (CH), which acts as the scheduler of cluster. The cluster members (CM) acquire and process a frame locally and send the results to the CH for data fusion in the current timestep. Subsequently, the CH will select some suitable camera nodes to be activated for the next tracking timestep. In order to ensure all camera nodes which sense the same target can communicate well with each other, we suppose synchronized and delay-free communication with a range twice the sensing radius. Furthermore, we also assume that there is no communication loss and all communication links are reliable so that the information can be readily shared. The key symbols in this paper are summarized in Table 1.

### 2.2. Motion and Measurement Model

The considered motion model and measurement models for camera networks, in this paper, are similar to those used in [24]. A 4-dimensional vector, $x_{k}={\left[x_{k},{\dot{x}}_{k},y_{k},{\dot{y}}_{k}\right]}^{T}$, denotes the target state at discrete timestep *k*, where $\left(x_{k},y_{k}\right)$ is the current target position and $\left({\dot{x}}_{k},{\dot{y}}_{k}\right)$ is its current velocity. *δ* is the constant sampling time interval between two successive measurements. Thus, the mobile target is described by a 2-D nonlinear motion model with the discrete time dynamic state equation given as follows:

(1) $$\begin{array}{c} x_{k}=\left[\begin{array}{c} x_{k-1}+\delta {\dot{x}}_{k-1}+\frac{\delta^{2}}{2}{\ddot{x}}_{k-1}\\ {\dot{x}}_{k-1}+\delta {\ddot{x}}_{k-1}\\ y_{k-1}+\delta {\dot{y}}_{k-1}+\frac{\delta^{2}}{2}{\ddot{y}}_{k-1}\\ {\dot{y}}_{k-1}+\delta {\ddot{y}}_{k-1} \end{array}\right], \end{array}$$

where ${\ddot{x}}_{k-1}$ and ${\ddot{y}}_{k-1}$ represent the acceleration of target in *x* and *y* coordinates at timestep k−1. $\nabla_{k-1}={\left[{\ddot{x}}_{k-1}\;{\ddot{y}}_{k-1}\right]}^{T}$ is considered as the noise vector, modeled by the zero-mean independent and identically distributed (IID) white Gaussian with covariance matrix $Q_{a}=diag\left(\tilde{q_{x}},\tilde{q_{y}}\right)$. Subsequently, the motion model of target can further be written as [7]

(2) $$x_{k}=f\left(x_{k-1}\right)+w_{k-1}=Ax_{k-1}+w_{k-1},$$

where $w_{k-1}$ is the process noise vector at timestep k−1, assumed the IID white Gaussian with covariance matrix

$$Q=\left[\begin{array}{cccc} {\tilde{q_{x}}\delta }^{4}/4 & {\tilde{q_{x}}\delta }^{3}/2 & 0 & 0\\ {\tilde{q_{x}}\delta }^{3}/2 & {\tilde{q_{x}}\delta }^{2} & 0 & 0\\ 0 & 0 & {\tilde{q_{y}}\delta }^{4}/4 & {\tilde{q_{y}}\delta }^{3}/2\\ 0 & 0 & {\tilde{q_{y}}\delta }^{3}/2 & {\tilde{q_{y}}\delta }^{2} \end{array}\right],$$

and A is the state transition matrix:

$$A=\left[\begin{array}{cccc} 1 & \delta & 0 & 0\\ 0 & 1 & 0 & 0\\ 0 & 0 & 1 & \delta \\ 0 & 0 & 0 & 1 \end{array}\right].$$.

The measurements can be given by the pixel coordinates of the center of the target in the image plane of the camera and the time elapsed between the two successive measurements [24]. As the static cameras are previously calibrated, there exists a homography to calculate the object’s position on the ground plane. The measurement model can be defined as:

(3) $$z_{i,k}=\left[\begin{array}{c} x_{i,k}\\ y_{i,k} \end{array}\right]=h_{i}\left(x_{k}\right)+v_{k},$$

where $\left(x_{i,k},y_{i,k}\right)$ denotes the pixel coordinate of the target in the image plane of camera $c_{i}$ at timestep *k*, $v_{k}$ is the measurement noise, which is considered to be Gaussian with zero mean and covariance matrix $R=diag(\left[\sigma^{2},\sigma^{2}\right])$, and $h^{i}\left(x_{k}\right)$ is the the pixel coordinates based on the homography $H^{i}$ corresponding to camera $c_{i}$, which is given by

(4) $$h^{i}\left(x_{k}\right)=\left[\begin{array}{c} h_{1}^{i}\left(x_{k}\right)\\ h_{2}^{i}\left(x_{k}\right) \end{array}\right]=\left[\begin{array}{c} \frac{H_{11}^{i}x_{k}+H_{12}^{i}y_{k}+H_{13}^{i}}{H_{31}^{i}x_{k}+H_{32}^{i}y_{k}+H_{33}^{i}}\\ \frac{H_{21}^{i}x_{k}+H_{22}^{i}y_{k}+H_{23}^{i}}{H_{31}^{i}x_{k}+H_{32}^{i}y_{k}+H_{33}^{i}} \end{array}\right],$$

where $\left(h_{1}^{i},h_{2}^{i}\right)$ is the pixel coordinates of the target in the image plane of camera $c_{i}$ and the values $H_{11}^{i},\cdots ,H_{33}^{i}$ are the elements of homography matrix $H^{i}$. Since we assume static cameras, the homography of cameras do not change with time *k* and the moving target.

### 2.3. Camera Node Sensing Model

All camera sensors are assumed to have the same sensing range $R_{s}$ which depends on the imaging capabilities of the sensor and be put at the same height in the target areas. Typically, by projecting the 3D visual sensing cone of the camera $c_{i}$ onto the 2D field, the FOV of the camera $c_{i}$, denoted $\hbar_{i}$, can be approximated by a fan with radius $R_{s}$.

**Figure 3:**
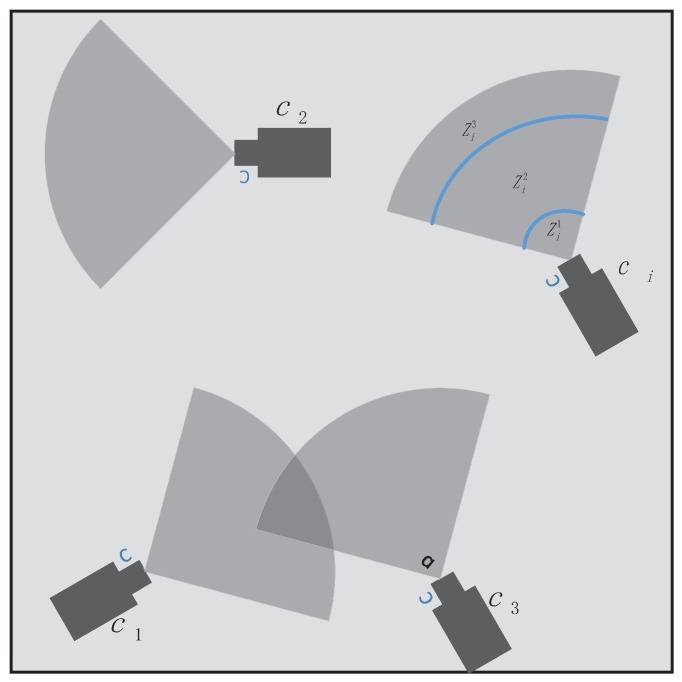
Sensing model in WCNs. Some camera sensors are deployed inside the surveillance area and $\hbar_{i}$ is a fan-shaped area with central angle $\alpha =90^{\circ }$. The zones of FOV of camera $c_{i}$, $Z_{i}=\left\{Z_{i}^{n};n=1,2,3\right\}$, are shown with different sized grey shapes. Target in $Z_{i}^{2}$ can be well viewed by $c_{i}$, but not well viewed in $Z_{i}^{1}$ and $Z_{i}^{3}$ because their distance is too close or too far.

In real scenarios, the surveillance area needs to be deployed with massive camera nodes to achieve good tracking results, but cheap cameras may have to be used to save money. As a result, those cheap cameras will not be able to well detect the target in every region of their FOV. See the illustration in Figure 3.

We assume that camera node $c_{i}$ can precisely view the target with the probability $\rho_{i}$. If the target position at timestep *k*, $T_{k}$, is inside the $Z_{i}^{1}$ which is close to the $c_{i}$, then $0<\rho^{\left(1\right)}<1$, because it is possible that only a little part of the target can be viewed by the $c_{i}$. Similarly, if the target is located inside the $Z_{i}^{3}$, the view of it in $c_{i}$ may be too little to be precisely sensed, thus $0<\rho^{\left(3\right)}<1$. Hence, the probability of the target being precisely sensed by $c_{i}$ yields

(5) $$\rho_{i}=\left\{\begin{array}{c} \rho^{\left(1\right)}<1\;\mathrm{if}\;T_{k}\in Z_{i}^{1}\\ \rho^{\left(2\right)}=1\;\mathrm{if}\;T_{k}\in Z_{i}^{2}\\ \rho^{\left(3\right)}<1\;\mathrm{if}\;T_{k}\in Z_{i}^{3}\\ 0\;\;\;\;\;\;\;\;\mathrm{if}\;T_{k}\notin Z_{i}, \end{array}\right.$$

and the relationship between the target and $Z_{i}$ is given by

(6) $$\left\{\begin{array}{cc} T_{k}\in Z_{i}^{1}\;\;\;\mathrm{if}\;d(c_{i},T_{k})\leq \zeta_{1}R_{s} & \&\;T_{k}\in \hbar_{i}\\ T_{k}\in Z_{i}^{2}\;\;\mathrm{if}\;\zeta_{1}R_{s}<d(c_{i},T_{k})\leq \zeta_{2}R_{s} & \&\;T_{k}\in \hbar_{i}\\ T_{k}\in Z_{i}^{3}\;\;\;\mathrm{if}\;\zeta_{2}R_{s}<d(c_{i},T_{k})\leq R_{s} & \&\;T_{k}\in \hbar_{i}\\ T_{k}\notin Z_{i} & \mathrm{if}\;T_{k}\notin \hbar_{i}, \end{array}\right.$$

where $d(c_{i},T_{k})$ is the distance between node $c_{i}$ and target $T_{k}$ and $0<\zeta_{1}<\zeta_{2}<1$.

To be clear, the probability of the target being accurately sensed by node $c_{i}$, $\rho_{i}$, is only taken into consideration in cluster nodes and cluster head selection phases to acquire a better cluster, which will be described in the corresponding sections. However, once a camera node is activated as a cluster member or cluster head, we deem that its measurements are accurate and reliable everywhere in its FOV.

### 2.4. Energy Consumption Model

The proposed energy consumption model is based on the power and activation times of a three-state model [25]. Cluster nodes, in active state, always have their cameras and radio on to acquire and process information data about the target and transmit or receive data between themselves and their neighbours, which results in most of the energy consumption. Sensor nodes in the alert state could receive data from their neighbours and periodically acquire and process data. Meanwhile, sensor nodes spend most of their time in sleep state during which nodes only periodically sense the target, which consumes the least energy among the three states [23].

Some symbols are necessary to model the energy consumption of camera nodes in these three states. Similar to [26,27], we suppose *a* joule (J) to be the energy expenditure for a camera node acquiring a frame and each frame processing produce, on average, $b_{t}$ bits for transmission, *p* is the average energy in joule (J) required for processing and producing 1 bit of information to be transmitted, and *t* and *r* is the average energy in joule (J) consuming in transmitting and receiving 1 bit, respectively.

(1) Active state. Let $E_{i}^{as}$ denote the energy consumption of node $c_{i}$ in active state. According to previous descriptions, the nodes in active state will acquire, process, receive and transmit data. If node $c_{i}$ is selected as CM, it will acquire and process the data about current target state, and then transmit its results to its CH. After receiving the data from the current CH, it also processes them locally and sends the packet including the processing results and the status data of $c_{i}$ to the CH. Suppose that the amount of the packet is also $b_{t}$. Then, the total energy consumption for $c_{i}$ at each timestep is
  (7) $$E_{i}^{as}=rb_{r}+a+2\left(p+t\right)b_{t},$$
  where $b_{r}$ is the average amount of the bits received from the CH. If node $c_{i}$ acts as the CH, besides the above actions, it needs to fuse different measurements from its CMs. Thus, its total energy cost yields
  (8) $$E_{ch}^{as}=2\left(rN_{a}+p\right)b_{t}+a+\left(u+t\right)b_{r},$$
  where $N_{a}$ is the number of its CMs and *u* is the average energy for fusing and producing 1 bit data.
(2) Alert state. Nodes in alert state could receive data from the current CH, process and transmit its results to the current CH. Therefore, the total energy cost for alert node $c_{j}$ becomes
  (9) $$E_{j}^{al}=rb_{r}+\left(p+t\right)b_{t}^{0},$$
  where $b_{t}^{0}$ is the number of data for $c_{j}$ to transmit.
(3) Sleep state. When a node is put into sleep state, most functions of the node are disabled. Only its sensing modules (e.g., camera sensor) could work periodically. To simplify system models, we assume that there is no energy consumption in the sleep state.

## 3. Decentralized SRCIF Algorithm for Measurement Fusion

In this section, we briefly introduce the process of the decentralized square root cubature information filter (SRCIF) algorithm which is used to fuse measurements from different sensor nodes. The SRCIF have many desirable properties compared to other filter algorithms, such that it is numerically stable and robust as well as easy to extend for multi-sensor state estimation [19]. For more theory details about the SRCIF, see [19,20,28].

The information filter are parametric RBFs, which uses the information matrix Y and information vector y at timestep *k*:

(10) $$Y_{k|k}={P_{k|k}}^{-1}$$

(11) $$y_{k|k}=Y_{k|k}x_{k|k}={P_{k|k}}^{-1}x_{k|k},$$

where P is the covariance matrix of the Gaussian distribution that represents the estimated state and x is the state vector of the target. Let $Y_{k|k}$ and $P_{k|k}$ be

(12) $$Y_{k|k}=S_{Y,k|k}S_{Y,k|k}^{T}$$

(13) $$P_{k|k}=S_{p,k|k}S_{p,k|k}^{T},$$

where $S_{Y,k|k}$ and $S_{p,k|k}$ are the triangular square-root matrixs of $Y_{k|k}$ and $P_{k|k}$, respectively. From Equations (10), (12) and (13), we can obtain that

(14) $$S_{Y,k|k}S_{Y,k|k}^{T}={\left(S_{p,k|k}S_{p,k|k}^{T}\right)}^{-1}=S_{p,k|k}^{-T}S_{p,k|k}^{-1}.$$

Hence, we get the relationship of square roots of the error covariance matrix and that of the information matrix

(15) $$S_{Y,k|k}=S_{p,k|k}^{-T}.$$

1. Prediction step of SRCIF
  (1) Compute the cubature points (i=1,2,⋯,2n) of timestep k−1
    (16) $${Ø}_{i,k-1|k-1}=x_{k-1|k-1}+S_{Y,k-1|k-1}\xi_{i}$$
    where $\xi_{i}$ is the i-th element of the following 2n cubature points set
    (17) $$\sqrt{n}\left[\left(\begin{array}{c} 1\\ 0\\ \vdots \\ 0 \end{array}\right),\cdots ,\left(\begin{array}{c} 0\\ 0\\ \vdots \\ 1 \end{array}\right),\left(\begin{array}{c} -1\\ 0\\ \vdots \\ 0 \end{array}\right),\cdots \left(\begin{array}{c} 0\\ 0\\ \vdots \\ -1 \end{array}\right)\right].$$
  (2) Compute the propagated cubature points (i=1,2,⋯,2n)
    (18) $${Ø}_{i,k|k-1}^{*}=f\left({Ø}_{i,k-1|k-1}\right).$$
  (3) Estimate the predicted state
    (19) $${\hat{x}}_{k|k-1}=\frac{1}{2n}\sum_{i=1}^{2n}{Ø}_{i,k|k-1}^{*}.$$
  (4) Estimate the square root factor of the predicted error covariance and information matrix
    (20) $$S_{p,k|k-1}=\mathrm{Tria}(\left[{ℵ}_{k|k-1}^{*}\;S_{Q}\right]),$$
    (21) $$S_{Y,k|k-1}={\left[{\left(S_{p,k|k-1}\right)}^{-1}\right]}^{T},$$
    where Tria denotes the operation of orthogonal triangular decomposition (for example, if S=Tria(A), then S is a lower-triangular matrix and $AA^{T}=SS^{T}$, see Section VI of [28] for more details about the operation of Tria), $S_{Q}$ is a square root of Q and
    (22) $${ℵ}_{k|k-1}^{*}=\frac{1}{\sqrt{2n}}\left[{Ø}_{1,k|k-1}^{*}-{\hat{x}}_{k|k-1}\;\;{Ø}_{2,k|k-1}^{*}-{\hat{x}}_{k|k-1}\;\;\cdots \;\;{Ø}_{2n,k|k-1}^{*}-{\hat{x}}_{k|k-1}\right].$$
  (5) Compute the predicted information state vector according to the Equation (11)
    (23) $${\hat{y}}_{k|k-1}=S_{Y,k|k-1}S_{Y,k|k-1}^{T}{\hat{x}}_{k|k-1}$$
2. Measurement update step of SRCIF for camera node $c_{j}$
  (1) Compute the cubature points (i=1,2,⋯,2n)
    (24) $${Ø}_{i,k|k-1}={\hat{x}}_{k|k-1}+S_{Y,k|k-1}\xi_{i}.$$
  (2) Compute the propagated cubature points (i=1,2,⋯,2n)
    (25) $$Z_{i,k|k-1}=h^{j}\left({Ø}_{i,k|k-1}\right).$$
  (3) Estimate the predicted measurement
    (26) $${\hat{z}}_{k|k-1}=\frac{1}{2n}\sum_{i=1}^{2n}Z_{i,k|k-1}.$$
  (4) Compute the weighted-centered matrices
    (27) $${ℵ}_{k|k-1}=\frac{1}{\sqrt{2n}}\left[{Ø}_{1,k|k-1}-{\hat{x}}_{k|k-1}\;{Ø}_{2,k|k-1}-{\hat{x}}_{k|k-1}\;\cdots \;{Ø}_{2n,k|k-1}-{\hat{x}}_{k|k-1}\right],$$
    (28) $${ℜ}_{k|k-1}=\frac{1}{\sqrt{2n}}\left[Z_{i,k|k-1}-{\hat{z}}_{k|k-1}\;Z_{2,k|k-1}-{\hat{z}}_{k|k-1}\;\cdots \;Z_{2n,k|k-1}-{\hat{z}}_{k|k-1}\right].$$
  (5) Estimate the cross-covariance matrix
    (29) $$\mathrm{Tria}(\left[\begin{array}{cc} {ℜ}_{k|k-1} & S_{R}\\ {ℵ}_{k|k-1} & 0 \end{array}\right])=T=\left[\begin{array}{cc} T_{11} & 0\\ T_{21} & T_{22} \end{array}\right],$$
    (30) $$P_{xz,k+1|k}=T_{21}{T_{11}}^{T},$$
    where $S_{R}$ is a square root of R, $T_{11}\in R^{m\times m}$ and $T_{22}\in R^{n\times n}$ are a lower-triangular matrix, and $T_{21}\in R^{n\times m}$ (m and n are the dimensions of measurement state and target motion state, respectively).
  (6) Evaluate the square-root information contribution matrix of sensor node $c_{j}$
    (31) $$S_{Y,k|k}^{\left(j\right)}=S_{Y,k|k-1}S_{Y,k|k-1}^{T}T_{21}{T_{11}}^{T}{\left(S_{R}^{-1}\right)}^{T}$$
  (7) Evaluate the information contribution vector and information contribution matrix of sensor node $c_{j}$
    (32) $$y_{k|k}^{\left(j\right)}=S_{Y,k|k}^{\left(j\right)}{\left(S_{R}^{-1}\right)}^{T}\left(z_{k}^{\left(j\right)}-{\hat{z}}_{k|k-1}\right)+S_{Y,k|k}^{\left(j\right)}{\left(S_{Y,k|k}^{\left(j\right)}\right)}^{T}{\hat{x}}_{k|k-1},$$
    (33) $$Y_{k|k}^{\left(j\right)}=S_{Y,k|k}^{j}{\left(S_{Y,k|k}^{j}\right)}^{T},$$
    where $z_{k}^{\left(j\right)}$ is the target measurement of $c_{j}$ at timestep *k*.

For a decentralized multi-camera tracking network, suppose $N_{c}$ camera nodes track the same target at timestep *k*. After sensing and processing locally, the nodes will transmit their results (including $y_{k|k}^{\left(i\right)}$ and $S_{Y,k|k}^{\left(i\right)}$ ) to a local fusion central (FC) simultaneously. Subsequently, the FC node will fuse these results with its own as follows:

(34) $${\hat{y}}_{k|k}={\hat{y}}_{k|k-1}+\sum_{i=1}^{N_{c}}y_{k|k}^{\left(i\right)},$$

(35) $$S_{Y,k|k}=\mathrm{Tria}\left(\left[S_{Y,k|k-1}\;S_{Y,k|k}^{\left(1\right)}\;\cdots \;S_{Y,k|k}^{\left(N_{c}\right)}\right]\right).$$

After obtaining the updated information vector $y_{k|k}$ and the updated information matrix $Y_{k|k}$, the corresponding error covariance matrix $P_{k|k}$ and the update target state yield

(36) $$P_{k|k}=Y_{k|k}^{-1}={\left(S_{Y,k|k}S_{Y,k|k}^{T}\right)}^{-1},$$

(37) $${\hat{x}}_{k|k}=P_{k|k}{\hat{Y}}_{k|k}.$$

According to the above descriptions, the CH computes ${\hat{y}}_{k|k}$ and $S_{Y,k|k}$ by briefly adding $y_{k|k}^{\left(i\right)}$, $S_{Y,k|k}^{\left(i\right)}$ from all CMs with the contributions of its own. The CMs only require us to execute the update step of SRCIF. Apart from gathering and integrating different measurements, the CH computes the predicted next state of target and then sends the state to the next cluster nodes. Therefore, the decentralized SRCIF algorithm could integrate the information data in an arbitrary order and distribute the computational burden of the measurements update among all the cluster nodes, which can be easily extended for multi-sensor fusion. Additionally, in the cluster-based tracking network using the decentralized SRCIF, in contrast to the consensus filter, only the CH requires us to receive the data from its CMs and the CMs do not need to communicate with other neighbours, which could heavily reduce the energy consumption resulting in extending the lifespan of the sensor network.

## 4. Selection of Task Cluster Nodes

In WCNs, each camera node usually has limited bandwidth and energy resources. Additionally, not all camera nodes that view the target contribute equally to detecting and tracking the target. Even if a node contributes a lot, it consumes too much energy to work well afterwards. Therefore, to increase the lifetime of a tracking network, only some camera nodes should be activated to act as task cluster nodes and some activated nodes should be deactivated to other states. However, this may lead to a decrease of tracking accuracy compared with traditional methods such that all camera nodes that view the target are included to integrate as many measurements as possible. Thus, an appropriate task cluster node selection mechanism should be put forward to balance the tracking accuracy and network lifetime.

In this section, we present our cluster node activation mechanism which adopts a greedy on-line decision approach to decide the most suitable task nodes. A camera node will be activated as a CM or deactivated according to both its measurements and its energy consumption. Therefore, an online decision mechanism that maximizes the trade-off between information gain and energy consumption is adopted. Let $\Gamma_{k+1}$ be a set of camera nodes that view the target at current timestep *k*: $\Gamma_{k+1}=\left\{c_{i}:c_{i}\in C,1\leq i\leq N_{c}\right\}$. The size of $\Gamma_{k+1}$ is $N_{c}$ which meets $0\leq N_{c}\leq N$. In this work, $\Gamma_{k+1}$ is considered as the set of candidate camera node at timestep k+1, which contains all current cluster nodes and all alert nodes at timestep *k*. Let $G_{i}$ be the information gain deriving from the measurements of $c_{i}$ and $C_{i}$ denote its energy consumption if it is activated. Subsequently, the expected contribution decision (CD) for a candidate node $c_{i}$ at timestep k+1 can be expressed as

(38) $$D_{i,k+1}=\alpha_{i,k+1}G_{i,k+1}-\beta_{i,k+1}C_{i,k+1},$$

where $\alpha_{i,k+1}$ and $\beta_{i,k+1}$ are weighting factors of expected information gain and energy consumption corresponding to camera $c_{i}$ at timestep k+1, respectively. Hence, for each node in $\Gamma_{k+1}$, we calculate its expected CD, and then rank all candidate camera nodes in descending order according to their CDs. Finally, some top-ranked camera nodes will be selected from the candidate set to be activated to form a new cluster for timestep k+1.

Then the expected information gain of $c_{i}$, $G_{i,k+1}$, and its weighting factor $\alpha_{i,k+1}$ are firstly computed. Different metrics have been proposed to gauge the tracking performance of the information filter [7,14,22,23]. Among them, the trace (sum of diagonal elements) of the predicted information matrix $Y_{k+1}$ computed at timestep *k* using Equation (33) corresponds to the mean squared error (MSE) of the updated state. Thus, it can be used to measure the expected information gain. Hence, the expected information gain of camera node $c_{i}$ at timestep k+1 can be given by

(39) $$G_{i,k+1}^{0}=tr\left(Y_{i,k+1}\right),$$

where tr(·) denotes trace operation. For facilitating data analysis and comparisons, we carry on the normalization calculation to the information gain as follows:

(40) $$G_{i,k+1}=\left(G_{i,k+1}^{0}-G_{min}^{0}\left(k+1\right)\right)/\left(G_{max}^{0}\left(k+1\right)-G_{min}^{0}\left(k+1\right)\right),$$

where $G_{min}^{0}\left(k+1\right)$ and $G_{max}^{0}\left(k+1\right)$ are minimum and maximum expected information gain values of candidate nodes, respectively. In general, a lager $G_{i,k+1}$ value implies more useful information gained by the measurements of $c_{i}$. In a camera sensor network with limited energy, trace is a linear metric. Therefore, in the case of our decentralized SRCIF, the CH can simply compute the predicted information gain of each candidate node resulting in a low burden to the limited-resource camera sensor network.

With respect to the weighting factor $\alpha_{i,k+1}$, we set it with different values based on both the predicted position of the target and the FOV of the camera $c_{i}$. According to Section 2.3, when the target is located in different zones of FOV of the camera $c_{i}$, the probability of the target being precisely sensed by the camera $c_{i}$, $\rho_{i}$, is different (see Equation (5)). Then the credibility of the information gain from $c_{i}$ should be also different. Thus, the weighting factor of the camera node $c_{i}$ at timestep k+1, $\alpha_{i,k+1}$, is given by

(41) $$\alpha_{i,k+1}=\rho_{i}.$$

Next, $\beta_{i,k+1}$ and $C_{i,k+1}$ for camera $c_{i}$ at timestep k+1 are calculated. Energy has been considered as the main resource in battery-powered WCNs. Thus, in this work, we only consider energy consumption as the resource’s consumption. Clearly, a camera node will keep an active state when it is a CM or CH. Therefore, the predicted energy consumption for camera $c_{i}$ at timestep k+1, $C_{i,k+1}$, is expressed as follows:

(42) $$C_{i,k+1}=E_{i}^{as},$$

where $E_{i}^{as}$ is the total energy consumption of active node $c_{i}$ during one tracking, computed by Equation (7). Note that $c_{i}$ cannot be selected as a CM, if its current remaining energy $E_{i}^{c}\leq C_{i,k+1}$.

$\beta_{i,k+1}$ weights the relative importance between predicted energy consumption $C_{i,k+1}$ and expected information gain $G_{i,k+1}$ for $c_{i}$ at timestep k+1. In traditional schemes, the absolute quantity of energy usually conducts as an indicate of *β* (like in work [23]). However, in this work, we set $\beta_{i,k+1}$ dynamically according to the current remaining energy of itself and other candidate nodes. Ordinarily, the importance of energy for a node varies depending on the current remaining energy of itself and others. The higher remaining energy a camera node has, the less importance the energy will be. Thus we adopt the relative amount of energy as an indicate, which is described as follows:

(43) $$\beta_{i,k+1}=a\exp\left({\bar{e}}_{k+1}^{0}-e_{i,k+1}^{0}\right),$$

where *a* is a constant factor, $e_{i,k+1}^{0}$ is normalized predicted remaining energy of $c_{i}$ and ${\bar{e}}_{k+1}^{0}$ is the average normalized predicted remaining energy of candidate nodes at timestep k+1. The normalization process of remaining energy is the same as that in the information gain. As shown in Equation (43), the camera nodes with higher remaining energy will be assigned a smaller weighting factor of energy consumption than those with lower remaining energy.

Note that the selection processes are conducted in the current CH and all candidate nodes just need to send their information data packets (including their positions, current energy, trace of predicted information matrix and relationship with the ${\hat{T}}_{k+1}$) to their CH separately. The CH does not require any knowledge about candidate nodes in advance, in contrast to that in [7]. Therefore, this mechanism is suitable for decentralized implementation of WCNs. Algorithm 1 summarizes the proposed mechanism for selection of cluster nodes at the end of timestep *k*.
**Algorithm 1.** The greedy on-line cluster node selection mechanism (operate at current CH)**Input:**
$\Gamma_{k+1}$, $E_{c}$, $\left\{\rho_{i};i\in \Gamma_{k+1}\right\}$, $N_{a}$. **Output:** A camera node set $\ell_{k+1}$ that contains $N_{a}$ top-ranked nodes based on their CDs.1.**for** each camera node $c_{i}\in \Gamma_{k+1}$2. Compute $\alpha_{i,k+1}$ using Equation (41)3. **if** $\rho_{i}>0$ and $e_{i}>E_{i}^{as}$4.  Compute expected $G_{i,k+1}^{0}$ using Equation (39).5.  $e_{i,k+1}\leftarrow e_{i}$.6. **else**7.  $G_{i,k+1}^{0}=0$, $e_{i,k+1}=0$.8. **end if**9.**end for**10.Normalize $\left\{e_{i,k+1}\neq 0;c_{i}\in \Gamma_{k+1}\right\}$ and $\left\{G_{i,k+1}^{0}\neq 0;c_{i}\in \Gamma_{k+1}\right\}$, then get set $\left\{e_{i,k+1}^{0}\right\}$ and set $\left\{G_{i,k+1}\right\}$.11.**for** Each camera node $c_{i}\in \Gamma_{k+1}$12. **if** $G_{i,k+1}^{0}\neq 0$13.  Compute $\beta_{i,k+1}$ using in Equation (43).14.  Compute its expected contribution decision $D_{i,k+1}$ using Equation (38)15. **else**16.  $D_{i,k}=-10,000$.17. **end if**18.**end for**19.Sort the set $D_{k}=\left\{D_{i,k};c_{i}\in \Gamma_{k}\right\}$ in the descending order, and then get a new set $\ell_{k+1}$ that contains $N_{a}$ top-ranked nodes based on expected contribution decision *D*.

## 5. Selection of CH

Dynamic cluster formation requires camera nodes to take time-varying states for enabling decentralized and determining optimal decision where the CH acts as the scheduler. A new CH for the next timestep is selected at the end of the current timestep. It communicates with its member nodes to exchange information data, gathers, fuses the data and then determines the next cluster (including CH and CMs). Hence, tracking performance strongly depends on which node acts as CH. Our objective is to select a reliable and efficient CH.

According to Section 2.4, the CH requires the most expenditure of energy among the task cluster nodes. Hence, selecting an appropriate CH could balance the remaining energy distribution of the current cluster. Furthermore, the smaller energy balance metric of the network implies a longer lifetime of the tracking network, given a total amount of energy [4]. Additionally, the situation that the selected CH could not view the target is not our intention but it happens occasionally in WCNs, since there may exit a great difference between the predicted and true target positions. Therefore, we cast such a selection problem as an optimization problem based on the predicted target position and the remaining energy distribution, to determine the CH $c_{k+1}^{h}$ as:

(44) $$\left\{\begin{array}{c} c_{k+1}^{h}=\underset{\forall c_{i}}{argmax}\;\psi \left(c_{i}\right)\\ \;\;\;\;\;s.t.\;\;\;e_{i}\geq E_{ch}^{as}\\ \;\;\;\;{\hat{T}}_{k+1}\in Z_{i}^{2}\\ \;\;\;\;c_{i}\in \ell_{k+1} \end{array}\right.$$

where $\psi (c_{i})$ is a weighted combination of $d(c_{i},{\hat{T}}_{k+1})$ and current remaining energy $e_{i}$ for camera node $c_{i}$. We define the weighted combination of $c_{i}$ as:

(45) $$\psi \left(c_{i}\right)=\theta \psi_{e}\left(e_{i}\right)+\left(1-\theta \right)\psi_{d}\left(d\left(c_{i},{\hat{T}}_{k+1}\right)\right),$$

where

(46) $$\psi_{e}\left(e_{i}\right)=\left(e_{i}-e_{i}^{min}\right)/\left(e_{i}^{max}-e_{i}^{min}\right);$$

(47) $$\psi_{d}\left(c_{i}\right)=\left(d^{max}\left(c_{i},{\hat{T}}_{k+1}\right)-d\left(c_{i},{\hat{T}}_{k+1}\right)\right)/\left(d^{max}\left(c_{i},{\hat{T}}_{k+1}\right)-d^{min}\left(c_{i},{\hat{T}}_{k+1}\right)\right);$$

θ∈[0,1] weights the energy priority; $d(c_{i},{\hat{T}}_{k+1})$ is the camera-target distance; $e_{i}^{max}=max\left\{e_{i};c_{i}\in \ell_{k+1}\right\}$; $e_{i}^{min}=min\left\{e_{i};c_{i}\in \ell_{k+1}\right\}$; $d^{max}\left(c_{i},{\hat{T}}_{k+1}\right))=max\left\{d\left(c_{i},{\hat{T}}_{k+1}\right);c_{i}\in \ell_{k+1}\right\}$ and $d^{min}\left(c_{i},{\hat{T}}_{k+1}\right))=min\left\{d\left(c_{i},{\hat{T}}_{k+1}\right);c_{i}\in \ell_{k+1}\right\}$. There are three restrictive conditions in Equation (44). The first condition requires the remaining energy of node selected as CH must has more than the least energy consumption of the CH during one tracking. The second condition requires the predicted target to be located in zone $Z_{i}^{2}$ of the camera $c_{i}$ if the camera node $c_{i}$ is selected as the CH. Finally, the third condition requires the candidate CHs to belong to the cluster node set $\ell_{k+1}$.

Under the above descriptions, this mechanism prioritizes nodes near the predicted target position and with more remaining energy. The camera nodes with less energy are not likely to be selected as the CH, which can delay their death and then lead to a longer network lifetime. Moreover, the nodes close to the predicted target position under the condition ${\hat{T}}_{k+1}\in Z_{i}^{2}$ could view the target well with a higher probability, given the error of the predicted target position. However, a candidate CH may not have both the most remaining energy and the shortest distance to the target at the same time. Hence, the goal of this CH selection mechanism is to make a good trade-off between the balanced remaining energy distribution and the robust tracking ability. Each camera node $c_{i}$ computes individually $d(c_{i},{\hat{T}}_{k+1})$ and remaining energy $e_{i}$, then sends them to the CH. Therefore, the CH does not require any knowledge about the candidate camera nodes in advance: nodes transmit to the CH everything the CH needs. Thus it is also efficient and suitable for decentralized implementation in WCNs. Algorithm 2 summarizes the proposed mechanism for selection of CH at the end of timestep *k*.
**Algorithm 2.** Selection of CH based weighted combination (operate in current CH)**Input:** Task cluster node set $\ell_{k+1}$ and their energy set $E_{c}$ and positions, predicted target position ${\hat{T}}_{k+1}$. **Output:** The CH $c_{k+1}^{h}$.1.Compute the distance between the predicted target position and each camera node $c_{i}$, $d\left(c_{i},{\hat{T}}_{k+1}\right)),c_{i}\in \ell_{k+1}$.2.$e_{k}^{max}\leftarrow max\left(\left\{e_{i};c_{i}\in \ell_{k+1}\right\}\right)$, $e_{k}^{min}\leftarrow min\left(\left\{e_{i};c_{i}\in \ell_{k+1}\right\}\right)$, $d^{max}\left(c_{i},{\hat{T}}_{k+1}\right))\leftarrow max\left(\left\{d\left(c_{i},{\hat{T}}_{k+1}\right)\right);c_{i}\in \ell_{k+1}\right\})$, $d^{min}\left(c_{i},{\hat{T}}_{k+1}\right))\leftarrow min\left(\left\{d\left(c_{i},{\hat{T}}_{k+1}\right)\right);c_{i}\in \ell_{k+1}\right\})$.3.**for** each camera node $c_{i}\in \ell_{k+1}$4. **if** ${\hat{T}}_{k+1}\in Z_{i}^{2}$ and $e_{i}>E_{ch}^{as}$5.  Compute $\psi_{e}\left(e_{i}\right)$ as in Equation (46) and compute $\psi_{d}\left(c_{i}\right)$ as in Equation (47).6.  Compute $\psi (c_{i})$ using Equation (45).7. **else**8.  $\psi (c_{i})=0$.9. **end if**10.**end for**11.$c_{k+1}^{h}\leftarrow argmax\left(\left\{\psi \left(c_{i}\right);c_{i}\in \ell_{k+1}\right\}\right)$.

## 6. Efficient and Robust Decentralized Tracking Scheme

The proposed decentralized target tracking scheme is divided into three mechanisms, namely measurements fusion and state prediction, selection of task cluster nodes and selection of CH, which have been described in detail above. Next, we will integrate these mechanisms into a decentralized tracking scheme.

All camera nodes are assumed in a sleep mode initially, but periodically awake to detect the target. Once a target appears in the monitor region boundary, the boundary nodes that sense the target will activate themselves to execute the tracking task. Meanwhile, the first boundary node that senses the target will automatically become the CH by broadcasting its information to inform other active nodes. Subsequently, the first CH will select the next CH and CMs to form a task cluster.

When the timestep *k* is up, the CH and CMs all capture the image of the target and perform SRCIF algorithm locally. After acquiring efficient data, the CMs forward the data to the CH after a random-delayed time with the conflict detect mechanism, CSMA/CA. The CH then fuses the received data together with its local measurements to achieve a more accurate current state estimation of the target. Subsequently, the target state at timestep k+1 is predicted in the CH and sent to CMs and all alert nodes. Each node $c_{i}\in \Gamma_{k+1}$ will calculate its trace of the predicted information matrix and send it with its current state data to the CH. Finally, the CH selects the next CMs and CH based on the received data. Note that if a CM selected by the last CH could not view the current target, it will fall into sleep state. However, if the selected CH could not view the current target yet, it will continue working and fuse different measurements without its own data. The concrete operations of the CH are described in detail in Algorithm 3, and the corresponding operations of the CMs and alert nodes are presented in Algorithm 4.
**Algorithm 3.** Operations in the CH at timestep *k***Start:** The data ${\hat{x}}_{k|k-1}$ and $S_{Y,k|k-1}$ which are from the last CH at timestep k−1.When the pre-set time is out, obtain the measurement $z_{k}^{ch}$, compute the $y_{k|k}^{\left(ch\right)}$ and $Y_{k|k}^{\left(ch\right)}$ based on the measurement update of the SRCIF as described in Section 3.Receive the packets with $<y_{k|k}^{\left(i\right)},Y_{k|k}^{\left(i\right)}>$ from each CMs, and then perform the information fusion to achieve ${\hat{y}}_{k|k}$ and $S_{Y,k|k}$ as in Equations (34) and (35).Compute $P_{k|k}$ and ${\hat{x}}_{k|k}$ as in Equations (36) and (37).Compute ${\hat{x}}_{k+1|k}$ and $S_{Y,k+1|k}$ based on the prediction step of the SRCIF as described in Section 3.Broadcast the packets with $<{\hat{x}}_{k+1|k},S_{Y,k+1|k}>$ to its CMs and all alert nodes.Compute its predicted ${\hat{Y}}_{k+1|k+1}^{ch}$ based on the measurement update phase of the SRCIF as described in Section 3.Receive the packets with $<e_{j},\rho_{j},p_{j},tr\left({\hat{Y}}_{k+1|k+1}^{j}\right)>$ from each camera $c_{j}\in \Gamma_{k+1}$.Select the new task cluster nodes and a new CH from current cluster nodes and all alert nodes according to the description in Section 4 and Section 5.Announce the selected CMs and CH with packets that include $<{\hat{x}}_{k+1|k},S_{Y,k+1|k}>$.
**Algorithm 4.** Operations in CMs or alert nodes at timestep *k***Start:** The data ${\hat{x}}_{k|k-1}$ and $S_{Y,k|k-1}$ which are from the last CH at timestep k−1.1.**for** each camera node $c_{i}\in \Gamma_{k+1}$2. **if** $c_{i}\in \ell_{k}$3.  When the pre-set time is up, measure the current target and obtain the measurement $z_{k}^{i}$.4.  Compute the $y_{k|k}^{\left(i\right)}$ and $Y_{k|k}^{\left(i\right)}$ based on the measurement update phase of the SRCIF as described in Section 3.5.  Transmit the packets with $<y_{k|k}^{\left(i\right)},Y_{k|k}^{\left(i\right)}>$ to its CH.6. **end if**7. Upon receiving the packet with $<{\hat{x}}_{k+1|k},S_{Y,k+1|k}>$, compute its predicted $S_{Y,k+1|k+1}^{i}$ based on the measurement update phase of the SRCIF as described in Section 3.8. Transmit the packet with $<e_{i},\rho_{i},p_{i},tr\left({\hat{Y}}_{k+1|k+1}^{i}\right)>$ to the CH.9. $c_{i}$ will keep active state if selected as a CM or CH, or it falls into sleep state.10.**end for**

## 7. Simulation Results and Evaluation

In this section, we evaluate the proposed mechanisms and compare them with the state-of-the-art algorithms. To evaluate and analyze the performances of the proposed algorithms, the software MATLAB is used to simulate the tracking scenario.

### 7.1. Simulation Setup

In our simulation, we consider the tracking scenario as shown in Figure 1. A low-speed motion target (e.g., human, animal) moves in a 500 m × 500 m square area with coordinates from [−250,250] to [−250,250]. The motion of the target is modeled with Gaussian distributed acceleration as given in Section 2.2. The covariance matrix of motion process noise $Q_{a}$ and covariance matrix of measurement noise R are assumed to be diag(0.1,0.1) and diag(5,5), respectively. The area is covered by a dense sensor network that contains N = 8000 camera sensor nodes whose FOV is a fan-shaped area with sensing range $R_{s}=30$ m and central angle $\alpha =90^{\circ }$ as shown in Figure 3. Without loss of generality, the initial energy of each camera node distributes uniformly in [0,1](J), and the energy consumption model of camera nodes in different states has been described in Section 2.4. Moreover, it is assumed that there is no wireless transmission error when nodes communicate with each other. To simplify the simulation, we set the total timesteps of measurements in one tracking K=100 and the time interval between two successive measurements δ=1 s. The homography matrix values of each camera $H^{i}$, in our system, are taken from the camera $C_{6}$ of APIDIS dataset [29] whose value are:

(48) $$H^{i}=\left[\begin{array}{ccc} 1930.8939 & -89.8033 & -2,393,800\\ 117.2530 & 91.8121 & 1,022,700\\ 0.3485 & -0.8720 & 1971.8862 \end{array}\right].$$

**Figure 4:**
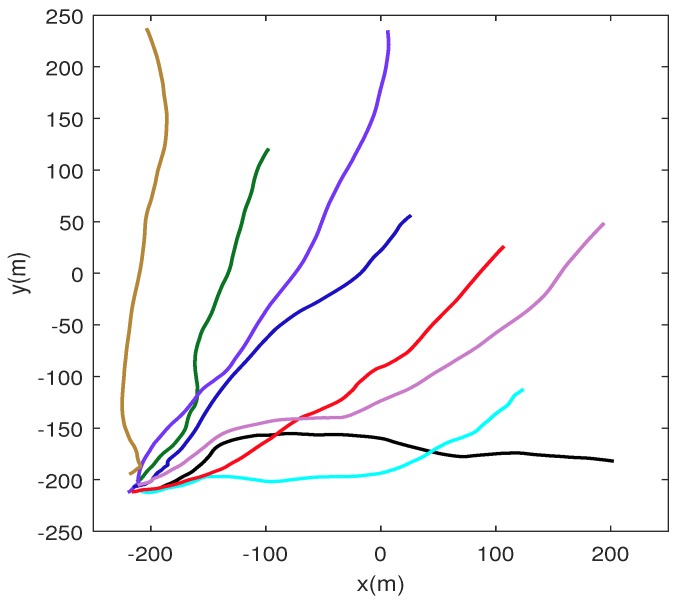
Some of the target trajectories which are used to test our methods in our simulated experiment. The trajectories are in solid lines with different colors, denoting different motion states of the target. To simplify the simulation, the spans of all trajectories are set within 100 s.

For a fair evaluation on each algorithm, we made a total of $N_{m}=1000$ independent Monte Carlo runs on each target trajectory. The results of the comparisons are averaged over 1000 different trajectories with different initializations. Table 2 summarizes other system parameters of this experimental environment which have been described in Section 2 with their settings and Figure 4 shows some of target trajectories used in our simulation experiment.

### 7.2. Decentralized SRCIF for Measurement Fusion

In this section, the accuracy and numerical robustness of EIF-, CIF- and SRCIF-based target tracking methods for the camera sensor network are compared under our proposed camera selection mechanisms.

With respect to the tracking accuracy comparison, the average root mean-squared error (ARMSE) in position is adopted as the indication of tracking accuracy, since it yields a combined measure of the bias and variance of a filter estimate [20]. The ARMSE in position is given by

(49) $$ARMSE\left[pos\right]=\sqrt{\frac{1}{N_{m}}\sum_{n=1}^{N_{m}}\frac{1}{K}\sum_{k=1}^{K}\left[{\left(x_{k,n}-x_{k}\right)}^{2}+{\left(y_{k,n}-y_{k}\right)}^{2}\right]},$$

where $\left(x_{k},y_{k}\right)$ is the true position of target at timestep *k* and $\left(x_{k,n},y_{k,n}\right)$ is the estimated target position in timestep *k* at *n*-th Monte Carlo simulation run. To check the numerical robustness of an information filter, we also adopt the filter divergence rate, as that used in [20]. The filter is declared to diverge when the average RMSE in the position of a Monte Carlo run (a tracking action) exceeds a given threshold $\partial_{0}$. In this simulation, we set $\partial_{0}$ to be 3.4.

**Figure 5:**
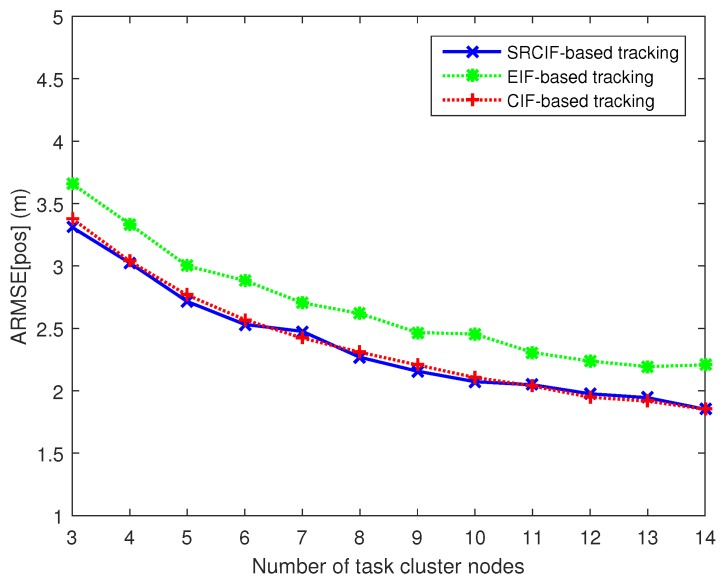
Position errors corresponding to different numbers of task cluster nodes in tracking based on different filter methods.

**Figure 6:**
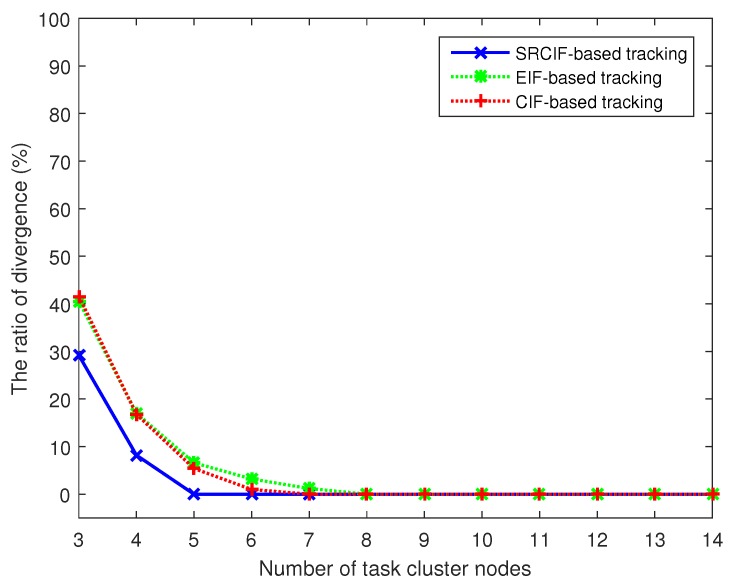
Ratio of divergence corresponding to different numbers of task cluster nodes in tracking based on different fusion methods.

As shown in Figure 5, the ARMSE in position for EIF-, CIF- and SRCIF-based target tracking decreases as the number of task cluster nodes $N_{a}$ increases. However, the improvement of tracking performance diminishes and seems to be trivial after $N_{a}>9$. In addition, Figure 5 also shows that both SRCIF and CIF consistently outperform EIF irrespective of $N_{a}$. The performance of CIF is almost identical to SRCIF, since the two are equally based on cubature filter. Figure 6 shows that the ratio of divergence occurs out of 1000 Monte Carlo runs corresponding to different number of task cluster nodes. The filter divergence rate of the three algorithms all decrease as $N_{a}$ increases which can be seen from Figure 6. However, EIF diverges most ($N_{a}<8$) and CIF diverges many times, especially when $N_{a}$ is less than 7. Unlike CIF and EIF, SRCIF only diverges when $N_{a}$ is 3 and 4. Table 3 summarizes the features of the three methods. From Figure 5 and Figure 6 and Table 3, it can be seen that SRCIF is superior to other filters considering both the tracking accuracy and numerical robustness.

### 7.3. Evaluation Results and Analysis of the Cluster Node Selection Mechanism

In this section, the tracking accuracy and energy consumption of the proposed cluster node selection mechanism (Algorithm 1) are evaluated and analysed in comparison with other relevant camera activation mechanisms in target tracking. Note that all mechanisms adopt the decentralized SRCIF to fuse different measurements and the same cluster head selection method as described in Section 5.

- M1. The CH activates or deactivates a camera analyzing the usefulness of its measurements and the resources as given in [23], which also adopts an on-line decision making approach that maximizes the trade-off between sensing gain and resource consumption. This work considers the remaining energy of each node instead of the relative energy in current candidate cluster to weight the resource consumption. Additionally, all camera nodes for which the rewards overtake the cost will be activated.
- M2. A fixed subset of camera nodes in the task cluster transmit their local information metrics to the CH. The active camera nodes are selected on the basis of their remaining energy.
- M3. The CH fuses the measurements from all camera nodes that could sense the current target in the current timestep [24].

**Figure 7:**
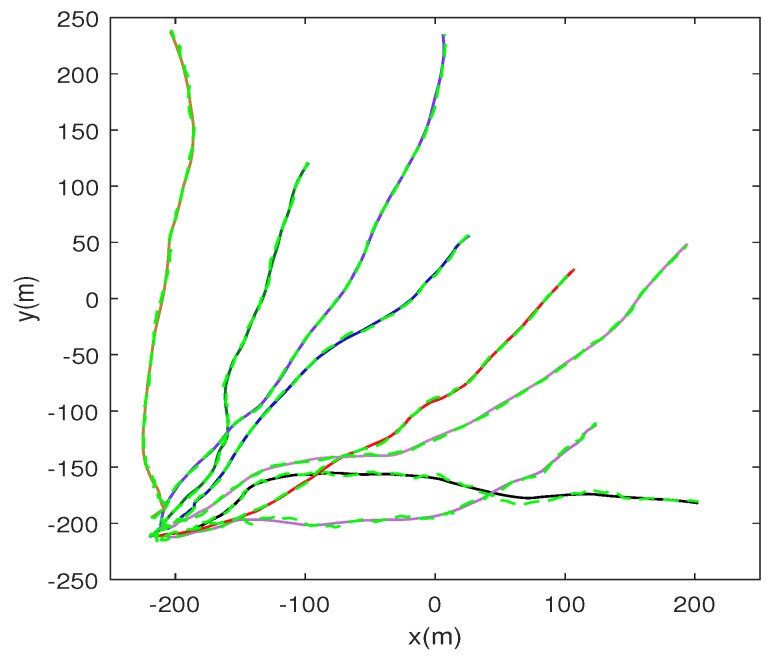
Tracking performance in some different trajectories of the target using our proposed node scheduling mechanism (Algorithm 1). The true target trajectories are shown in a solid line with different colors and the estimated trajectories are shown in dashed lines with green.

From Figure 5, it can be seen that the improvement of tracking performance diminishes and seems to be trivial after $N_{a}>9$. Thus, in this simulation experiment, we fix the number of task cluster nodes in Algorithm 1 as well as in M2, $N_{a}=9$. Figure 7 shows the tracking results under different target trajectories when using our node scheduling mechanism (Algorithm 1). The true trajectories of target are in a solid line with different colors and all the estimated target trajectories are in dashed with green. From Figure 7, it can be seen that the Algorithm 1 performs well in the estimation of target state under different target trajectories.

**Figure 8:**
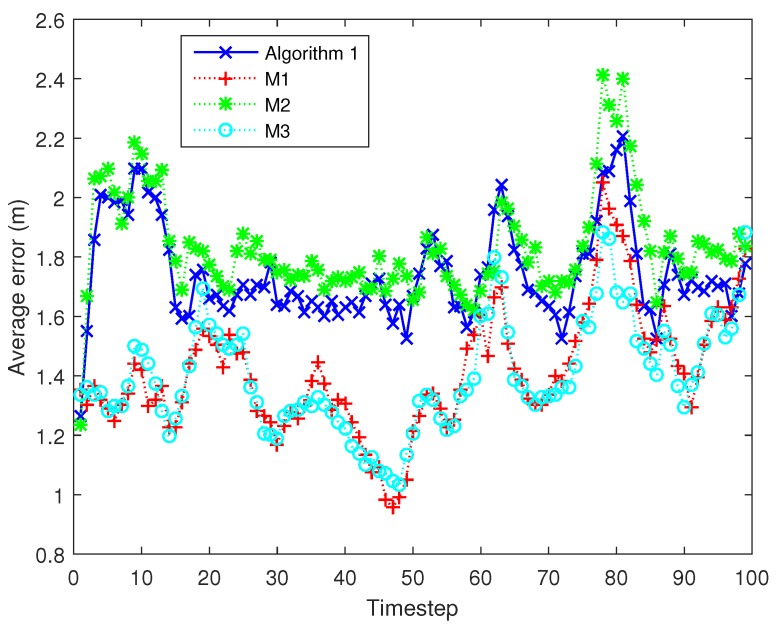
Averaged tracking error of different algorithms at different timesteps. The tracking errors are averaged over 1000 independent Monte Carlo runs under the same target trajectories.

**Figure 9:**
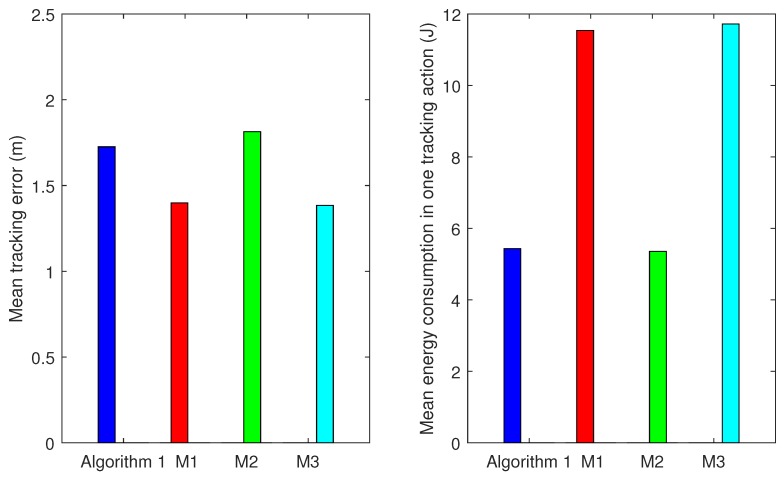
Comparison between Algorithm 1, M1, M2 and M3: the left figure depicts the mean tracking error in one timestep and the right one depicts the mean energy consumption in one tracking action.

**Figure 10:**
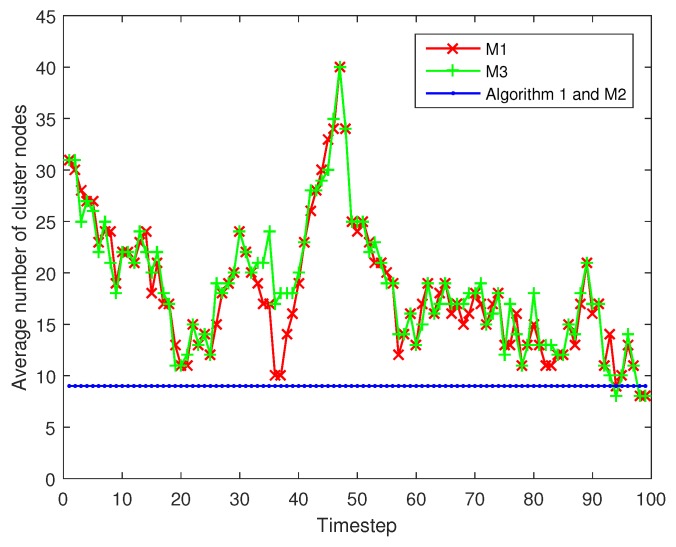
Average number of task cluster nodes of different methods at different timesteps under the same target trajectory.

Next, the Algorithm 1 is compared with above mechanisms M1, M2 and M3 under the same trajectories. Figure 8 shows the averaged error at different timesteps using different camera node scheduling mechanisms. From Figure 9, we can find that M3 and M1 achieve the lowest mean tracking error in one timestep (1.3839 and 1.3984 m, respectively) but the highest energy consumption in one tracking action (11.7215 and 11.5384 J, respectively). The reason for this is that M3 and M1 integrate a large number of measurements which are transmitted by their numerous cluster nodes (shown in Figure 10). The mean tracking error of Algorithm 1 is 1.7257 m, higher than that of M3 and M1, but still suitable for most applications, while its mean energy consumption is the lowest (5.436 J), 53.6% lower than M3 and 52.8% lower than M1. M1 activates all nodes with positive gain-cost balance, which may lead to numerous task nodes and unnecessary energy consumption. Our camera node scheduling mechanism only selects $N_{a}$ top-ranked nodes based on their contribution decision. As for the comparison between M2 and our Algorithm 1, they are both with the same and fixed number of task cluster nodes ($N_{a}=9$). However, Algorithm 1 outperforms M2 in tracking accuracy: 1.7257 m against 1.8543 m with almost identical energy consumption, because it considers not only the remaining energy of a camera node but also its the information gain when selecting the next cluster nodes. Table 4 summarizes the features of Algorithm 1, M1, M2 and M3. Therefore, our method performs the best among the four algorithms in balancing the tracking accuracy and network lifetime.

### 7.4. Evaluation Results and Analysis of Cluster Head Selection

This section analyzes and compares the proposed CH selection method (Algorithm 2) with the following methods.

- C1. This method selects the active node that is closest to the estimated target position as the CH, as in [24].
- C2. In this method, an active camera node that has the highest remaining energy will be selected as the CH.

We compare our method with C1 and C2 from the perspective of the standard deviation of remaining energy and the ratio that the selected CH cannot view the target in a tracking action in a tracking action (100 timesteps). The standard deviation of the remaining energy in a task cluster could be adopted as the energy balance metric of the networks according to the work [4]. To compare fairly, all methods use the decentralized SRCIF and camera node activation method as described in Section 4.

**Figure 11:**
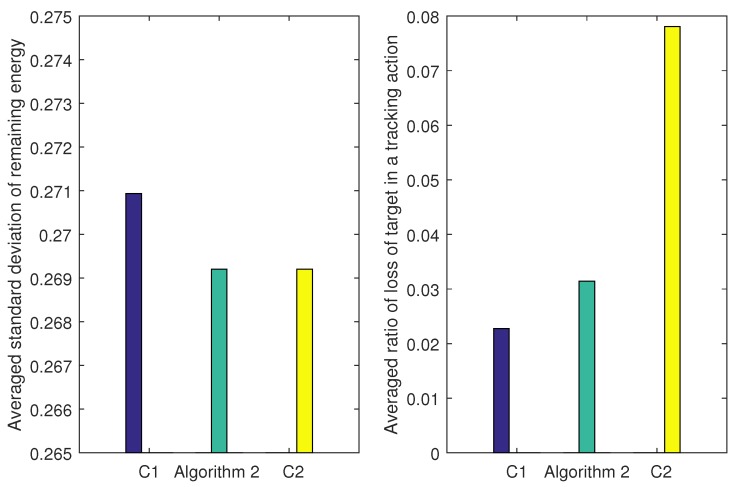
Comparison between Algorithm 2, C1 and C2: the left figure depicts the averaged standard deviation of the remaining energy in a task cluster and the right one depicts the averaged ratio of loss of target in a tracking action.

Figure 11 shows the comparison results and Table 5 summarizes their features. Algorithm 2 outperforms C2 by 60 percent in the ratio of loss of target: 0.0314 against 0.0780 with an almost identical standard deviation of the remaining energy. As for the comparison between C1 and Algorithm 2, the C1 and Algorithm 2 both achieve a good result in the ratio of loss of target in a tracking action: 0.0227 and 0.0314. However, Algorithm 2 outperforms C1 in energy balance metric of network, which may lead to a longer network lifetime. Therefore, Algorithm 2 makes the best trade-off between the balanced remaining energy distribution and the robust tracking ability.

It should be noted that under the assumptions in our simulation experiment, our proposed approach works well when the target speed is lower than 15 m/s. When the target speed is above 15 m/s, the scenario that only few of candidate nodes could view the current target will occur. Thus, the multi-sensor target tracking scheme using our approach will fail when the target moves too fast according to our simulation experiment.

## 8. Conclusions and Future Work

In this paper, we considered a cluster-based single target tracking situation in dense WCNs where the cluster will dynamically change with the moving of the target. Considering some characteristics and constraints of WCNs, an effective and robust decentralized tracking scheme is proposed in this paper to balance the energy consumption and tracking accuracy. The tracking scheme integrates different mechanisms: (1) a more realistic camera node sensing model; (2) a decentralized SRCIF for fusing different observation results; (3) a greedy on-line decision mechanism to select task cluster nodes; and (4) an efficient and stable mechanism for selection of cluster head (CH). The proposed scheme could distribute the computational burden to each cluster node. Furthermore, the computational burden performed by the cluster nodes (including the CH) is roughly constant regardless of the cluster size. Therefore, it can be easily extended for multi-sensor collaborative target tracking in WCNs. Additionally, the proposed scheme selects a fixed number of task cluster nodes based on the defined contribution decision (CD), which makes it applicable to a sensor network where each sensor node is resource-constrained. Each mechanism is evaluated and compared with related mechanisms using state of the art methods. The comparison results demonstrate that the proposed scheme behaves really well in balancing the resource consumption and tracking accuracy.

In our future endeavors, we aim to carry out our further work on the following aspects. The first aspect is to investigate the multi-target tracking schemes in WCNs, which will be more complicated than those in the single target scenario. Then, the recovery mechanism for cases of emergency or target loss in practice should also be discussed. The third valuable aspect is to investigate the problem of tracking a high-speed target in WCNs. In addition, the simulation of our approach is implemented via MATLAB platform, which is not applicable in practical wireless sensor network nodes where the advanced software is not available in MCU and embedded system. Therefore, one of our following tasks is the implementation of the solution in C language to use directly in sensor nodes. Finally, how to reduce the cost of building a tracking network is also a significant subject in our future work.

## Figures and Tables

**Table 1:** Key symbols and notations.

Symbol	Notation	Symbol	Notation
$x_{k}$	target state vector at timestep *k*	$w_{k}$	process noise vector at timestep *k*
Q	covariance matrix of $w_{k}$	$z_{k}^{i}$	measurement vector of $c_{i}$ at timestep *k*
$h_{i}$	measurement model of camera node $c_{i}$	R	covariance matrix of $v_{k}$
*f*	target motion model	$v_{k}$	measurement noise at timestep *k*
$H^{i}$	homography matrix of $c_{i}$	$\hbar_{i}$	the field of view (FOV) of $c_{i}$
$Z_{i}$	zones of FOV of camera $c_{i}$	$\rho_{i}$	sensing probability of target detected by $c_{i}$
$T_{k}$	position of target at timestep *k*	$\ell_{k}$	set of task cluster nodes at timestep *k*
$E_{i}^{as}$	energy consumption of $c_{i}$ (CM)	$E_{ch}^{as}$	energy consumption of cluster head
$E_{j}^{al}$	energy consumption of $c_{j}$ in alert state	P	covariance matrix of $x_{k}$
y	information vector	Y	information matrix
$S_{Y}$	triangular square-root matrix of Y	$S_{p}$	triangular square-root matrix of P
$\Gamma_{k}$	set of camera nodes that view the	$d(c_{i},T_{k})$	distance between node $c_{i}$ and target
	target at timestep *k*		at timestep *k*
$N_{c}$	number of camera nodes in $\Gamma_{k}$	$N_{a}$	number of camera nodes in $\ell_{k}$
*D*	contribution decision	*G*	normalized information gain
$C_{i}$	energy consumption	$e_{i}$	current remaining energy of $c_{i}$
*α*	weighting factor of information gain	*β*	weighting factor of energy consumption
*ψ*	weighted combination	$R_{s}$	sensing range
tr	trace operation	$p_{i}$	position of camera node $c_{i}$

**Table 2:** System parameters of simulation environment and their settings.

Description	Symbol	Setting
Sensing probability of node $c_{i}$ if the target is inside the $Z_{i}^{1}$	$\rho^{\left(1\right)}$	0.8
Sensing probability of node $c_{i}$ if the target is inside the $Z_{i}^{2}$	$\rho^{\left(2\right)}$	1
Sensing probability of node $c_{i}$ if the target is inside the $Z_{i}^{3}$	$\rho^{\left(3\right)}$	0.8
Weighting factor of energy priority	*θ*	0.7
Weighting factors of detection range	$\zeta_{1},\zeta_{2}$	0.1,0.9
Acquisition cost	*a*	$5.0\times 10^{-3}$ J
Processing cost	*p*	$4.4\times 10^{-8}$ J/bit
Fusing cost	*u*	$4.4\times 10^{-8}$ J/bit
Transmission cost	*t*	$2.2\times 10^{-7}$ J/bit
Receiving cost	*r*	$2.92\times 10^{-6}$ J/bit
Amount of transmission data(CM)	$b_{t}$	160 bits
Amount of transmission data(Alert)	$b_{t}^{0}$	100 bits
Amount of receiving data from CH	$b_{r}$	100 bits

**Table 3:** Features of different fusion algorithms.

Fusion Algorithms	Tracking Accuracy	Numerical Robustness
SRCIF-based tracking	high	high
CIF-based tracking	high	moderate
EIF-based tracking	low	low

**Table 4:** Features of different cluster node selection methods.

Node Selection Methods	Tracking Error	Energy Consumption
Algorithm 1	moderate (1.7257 m)	low (5.436 J)
M1	low (1.3984 m)	high (11.5384 J)
M2	high (1.8543 m)	low (5.484 J)
M3	low (1.3839 m)	high (11.7215 J)

**Table 5:** Features of different cluster head selection methods.

CH Selection Methods	Standard Deviation of Remaining Energy	Ratio of Loss of Target in a Tracking Action
Algorithm 2	low (0.269)	few (0.0314)
C1	high (0.271)	few (0.0227)
C2	low (0.269)	much (0.0780)
